# EHMTI-0392. Migraine and risk of ischemic heart disease: a systematic review and meta-analysis of observational studies

**DOI:** 10.1186/1129-2377-15-S1-J10

**Published:** 2014-09-18

**Authors:** S Sacco, R Ornello, P Ripa, F Pistoia, A Carolei

**Affiliations:** 1 Department of Applied Clinical Sciences and Biotechnology, University of L'Aquila, L'AQUILA, Italy; 2 Department of Biotechnological and Applied Clinical Sciences, University of L'Aquila, L'AQUILA, Italy

## Background

Several studies have assessed the possible increased risk of ischemic heart diseases in migraineurs, drawing different conclusions.

## Aim

To define and update the issue of the association between migraine and ischemic heart disease we performed a systematic review and meta-analysis of the available observational studies.

## Methods

Electronic databases were systematically searched up to April 2014 for observational studies dealing with the risk of any form of ischemic heart disease in subjects with migraine.

## Results

**Figure 1:**
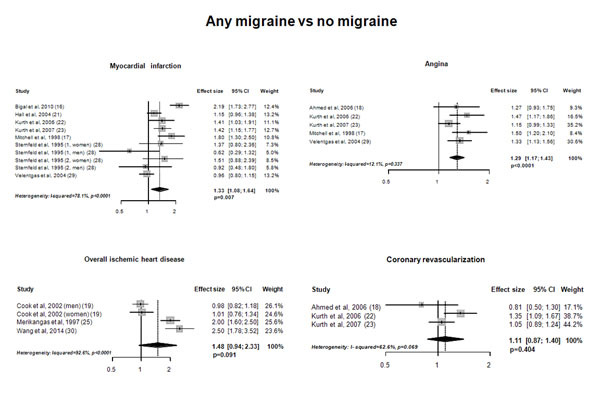


**Figure 2:**
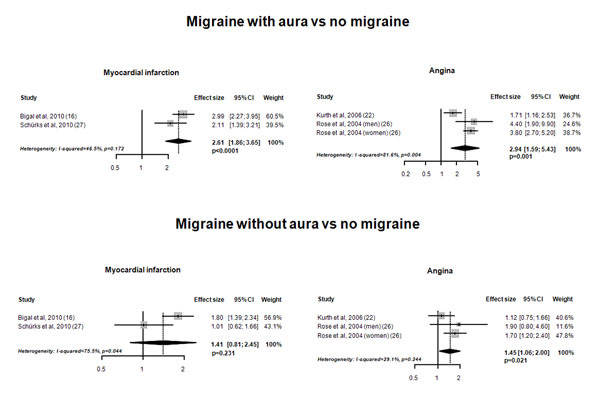


Out of 3,348 records, we identified 15 studies which were included in the meta-analysis. The pooled analysis indicated an increased risk of myocardial infarction (pooled adjusted effect estimate 1.33, 95% CI 1.08-1.64; P=0.007) and of angina (pooled adjusted effect estimate 1.29, 95% CI 1.17-1.43; P<0.0001) in subjects with any migraine compared to non-migraineurs (Figure 1); subjects with migraine with aura had an increased risk of myocardial infarction and of angina (Figure 2). At variance, the pooled analysis did not indicate an increased risk of ischemic heart disease or of coronary revascularization procedures in subjects with any migraine compared to non-migraineurs (Figure 1).

## Conclusions

Based on our data indicating an association of migraine with myocardial infarction and angina and on previous data showing an association of migraine, and particularly migraine with aura, with an increased risk for stroke, migraine can be appropriately considered an overall risk factor for cardiovascular diseases.

No conflict of interest.

